# Dissecting the alternation landscape of mitochondrial metabolism-related genes in lung adenocarcinoma and their latent mechanisms

**DOI:** 10.18632/aging.204803

**Published:** 2023-06-15

**Authors:** Xing Jin, Di Liu, Demiao Kong, Xiaojiang Zhou, Liken Zheng, Chuan Xu

**Affiliations:** 1 Department of Thoracic Surgery, Guizhou Provincial People’s Hospital, Guiyang, Guizhou, China; 2 Genecast Biotechnology, Wuxi, Jiangsu Province, China

**Keywords:** NSCLC, lung adenocarcinoma, mitochondrial metabolism-related genes, prognosis, mechanism

## Abstract

Lung adenocarcinoma (LUAD) is the most common histological subtype of lung cancer with high incidence and unsatisfactory prognosis. The majority of LUAD patients eventually succumb to local and/or distinct metastatic recurrence. Genomic research of LUAD has broadened our understanding of this disease’s biology and improved target therapies. However, the alternation landscape and characteristics of mitochondrial metabolism-related genes (MMRGs) in LUAD progression remain poorly understood. We performed a comprehensive analysis to identify the function and mechanism of MMRGs in LUAD based on the TCGA and GEO databases, which might offer therapeutic values for clinical researchers. Then, we figured out three hub prognosis-associated MMRGs (also termed as PMMRGs: ACOT11, ALDH2, and TXNRD1) that were engaged in the evolution of LUAD. To investigate the correlation between clinicopathological characteristics and MMRGs, we divided LUAD samples into two clusters (C1 and C2) based on key MMRGs. In addition, important pathways and the immune infiltration landscape affected by LUAD clusters were also delineated. Further, we nominated potential regulatory mechanisms underlying the MMRGs in LUAD development and progression. In conclusion, our integrative analysis enables a more comprehensive understanding of the mutation landscape of MMRGs in LUAD and provides an opportunity for more precise treatment.

## INTRODUCTION

Lung cancer (LC) accounts for the second predominant cause of cancer-related death worldwide, with 5-year overall survival rates of less than 20% [1, 2]. Lung adenocarcinoma (LUAD) is the most frequent pathological subtype of LC that constitutes about 60% of LC cases [3]. Furthermore, smoking is generally described as the most common risk factor for LUAD [4]. Other non-smoking associated risk factors include long-term exposure to radon, occupational exposure to carcinogens, and air pollution [5]. Despite the advent of comprehensive therapies and novel clinical drugs, therapeutic outcomes of patients with recurrence and metastatic adenocarcinoma remain poor [6].

The term “oncogene addiction” refers to the phenomenon in which tumorigenesis is dependent on the specific activity of a single oncogene [7]. The key oncogenic drivers in LUAD are mutations of TP53, EGFR, KRAS, and ALK rearrangements [8, 9]. In addition, novel molecular divers are emerging, such as BRAF mutations and HER2. A majority of oncogenic studies to date almost exclusively focused on the changes in nuclear genomics while ignoring the mitochondrial genome. Being the foundation for a wide range of inborn metabolic disorders, mitochondrial genome variation and translation are currently recognized as hallmarks of cancer progression [10, 11]. Specifically, gene expression alternations as a consequence of mitochondrial pathway abnormalities and metabolic dysregulation can lead to tumor invasion and metastasis as well as immune evasion [12]. And such mutations in mitochondrial genomes have been frequently corroborated to be involved in the malignancy progression of LUAD [13, 14].

With the rapid development of sequencing technologies, the complex genomic alterations in LUAD have been extensively deciphered over the past several decades. However, the roles and mechanisms of mitochondrial metabolism-related genes (MMRGs) in LUAD and their clinical significance remain primarily in a backwater inhabited by a few academics and professionals and are not visible to public researchers. For example, a study by Ye et al. showed that a signature model based on four mitochondrial energy metabolism pathway-associated genes could excellently diagnose patients with LUAD [15]. The limitation of this study was that it was a correlational study and did not explore the underlying molecular biological mechanisms and illustrate the potential of these genes in LUAD prevention and treatment. Further studies are required to understand the role of MMRGs in LUAD progression and their clinical significance.

The Cancer Genome Atlas (TCGA) is a collective effort conducted to decode the vast amounts of genomic data that drive diverse malignancies through large-scale sequencing [16]. Clinical trials based on expression profiling have been successfully applicated in predicting the prognosis of breast cancer and derivative large B-cell lymphomas, offering crucial information for treatment planning [17, 18]. The expression patterns of MMRGs in LUAD development and their corresponding clinical and mutational characteristics are relatively concordant across studies. In the present study, we investigated the role and latent mechanisms of crucial MMRGs, extracted from the TCGA and GEO database, during the development and progression of LUAD. Also, we explored the correlation of prognostic MMRGs (PMMRGs) in the evolution of LUAD. These findings may provide clues for the prevention and personalized target therapy of LUAD.

## MATERIALS AND METHODS

### Data preparation

Aiming to comprehensively analyze the roles of MMRGs during LUAD progression, the whole genome sequence, RNA sequencing (RNA-seq), microRNA-seq (miRNA-seq), DNA methylation data, and corresponding clinicopathological information were downloaded from the TCGA-LUAD database using UCSC XENA. For further investigation of our study, two datasets (GSE33479 and GSE4573) were obtained from the public GEO database (https://www.ncbi.nlm.nih.gov/geo/). GSE33479, a gene expression profile calculated by microarrays, was designed to figure out the expression difference between 24 normal and 14 LUAD samples, thus identifying latent MMRGs affecting LUAD development. Determined to filter those MMRGs engaged in LUAD survival (namely PMMRGs), GSE4573 was an RNA-seq dataset that comprised 130 LUAD samples.

### Clinical characterization of LUAD cluster identified by MMGRs

We performed genotyping analyses to determine the optimal conditions for LUAD samples clustering. Then, nonnegative matrix factorization (NMF), performed by R package NMF (0.26), was used to classify these LUAD samples into two clusters based on the gene expression profiles of MMRGs. Ten algorithms used for splitting the LUAD samples into two clusters were demonstrated as Ward, Single, Complete, Average, McQuitty, Median, Centroid, Kmeans, Marriot and trcovw. Kaplan-Meier analysis was exploited to investigate the correlation between two clusters and overall survival (OS), progression-free interval (PFI), disease-specific survival (DSS), and disease-free interval (DFI) of LUAD, respectively. Furthermore, we illustrated the relationship between clusters and clinicopathological parameters of LUAD. According to the clinical information from the TCGA-LUAD database, we separately explored the correlation between LUAD clusters and age, gender, radiotherapy and chemotherapy receiving, TNM stage, and tumor recurrence, respectively.

### Functional analysis of MMRGs

Based on the above LUAD clusters, the “limma” R package (3.52.4) was employed to ascertain the differential expression genes (DEGs) in the RNA-seq data. Simultaneously, |log2 fold change| >1.0 and adjusted p <0.05 were chosen as the criteria for screening differentially expressed MMRGs (DEMMRGs). Subsequently, we further illuminated the functional characteristics of DEMMRGs in LUAD progression. Gene Ontology (GO) is an internationally standardized database that describes gene products and gene functions across species and databases [19]. We used the “clusterProfiler” package (4.4.4) in R to perform GO-MF enrichment analysis of MMRGs to further investigate the major pathways through which these MMRGs participated in LUAD progression. Simultaneously, the GSVA algorithm was exploited to evaluate the immune infiltration score and tumor characteristic pathway score of LUAD patients, thereby analyzing the effect of genotyping of MMRGs on immune cells and tumor characteristic pathways.

### Analysis of latent regulatory mechanisms of MMRGs

In general, the expression level of MMRGs is subject to multiple factors. To understand the possible regulatory mechanisms, present in cluster-related MMRGs, we analyzed the latent mechanisms from three perspectives: gene mutation, DNA methylation, and transcription factor regulation. Based on the whole genome sequence from the TCGA-LUAD database, we distinguished the mutational MMRGs, thus investigating their alternations in the expression levels. The DNA methylation data of LUAD and RNA-seq data were used to observe the influence of methylation on MMRGs expression by co-expression analysis with R < -0.25 as a screening criterion. We determined the possible transcription factors targeted by the differentially expressed MMRGs based on the Chip-seq data from the ENCODE database. On the other hand, RNA-seq data were used to calculate the co-expression relationship between MMRGs and transcription factors, and the screening criterion of transcriptional regulation was defined as | R | >0.25.

### Statistical analysis

All statistical analyses and data visualization were conducted using R software (version 4.1.2). The data were presented as means ± standard deviations. Wilcoxon rank sum analysis was used to compare the MMRGs between different groups, and Kaplan-Meier analysis was tailored to evaluate the survival difference among LUAD patients in distinct groups. P value <0.05 was considered statistically significant.

## RESULTS

### Identification of DEMMRGs in LUAD

**Figure 1:**
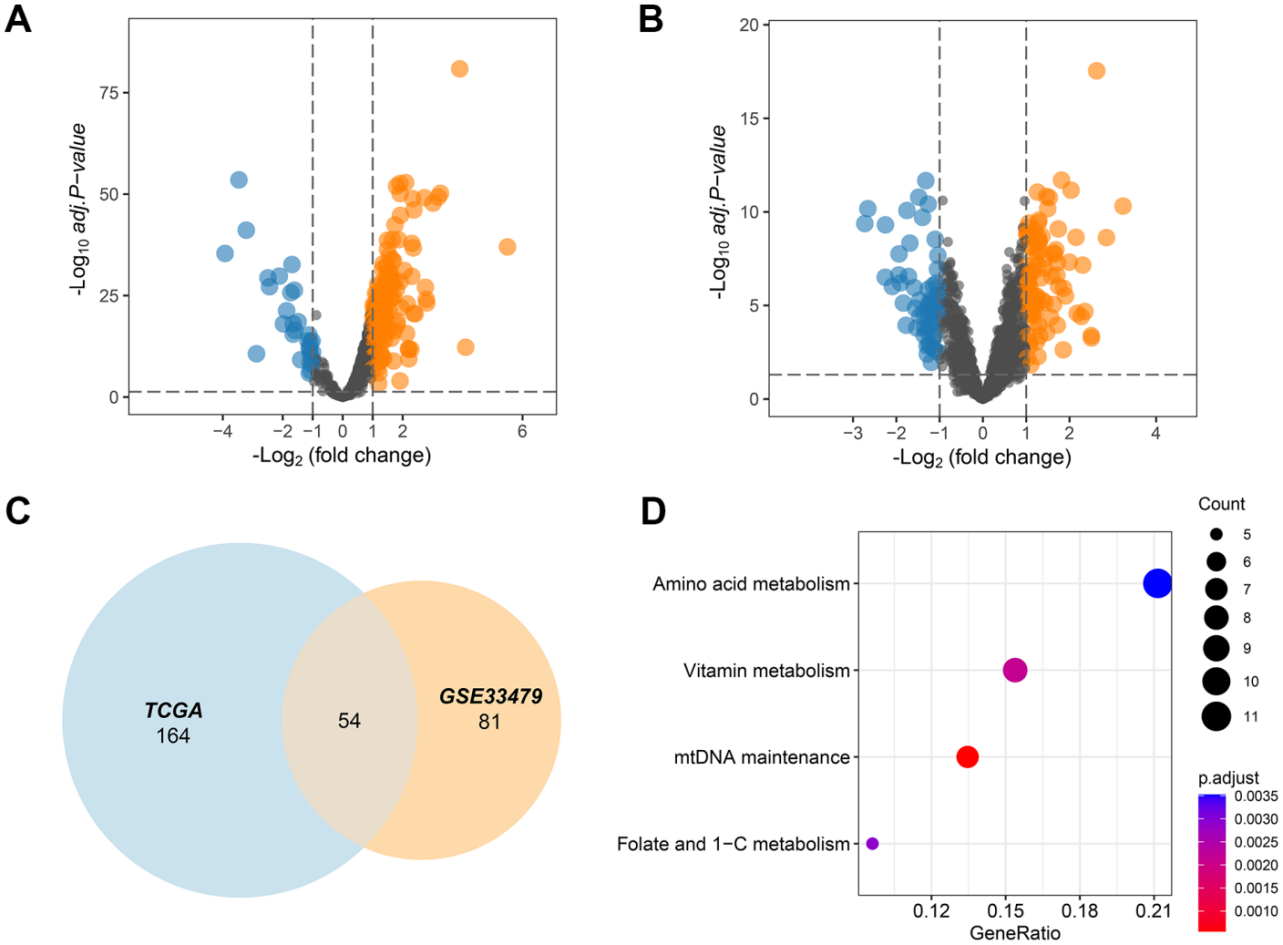
**MMRGs identification in LUAD.** (**A**, **B**) Volcano plots of the differentially expressed MMRGs in the TGCA and GSE33479 database. (**C**) Venn diagram of differentially expressed MMRGs in TGCA and GSE33479. (**D**) KEGG pathway enrichment analysis of 54 pivotal MMRGs.

The MITOCARTA3.0 (https://www.broadinstitute.org/mitocarta/mitocarta30-inventory-mammalian-mitochondrial-proteins-and-pathways) database collected 1136 key genes that played a crucial role in the functions of mitochondria. These 1136 mitochondrial genes were then screened by TCGA and GSE33479 dataset to select those deregulated MMRGs affecting LUAD progression. The results showed that 188 MMRGs were overexpressed and 30 MMRGs were low expressed in LUAD cases according to the TCGA database (Figure 1A). On the other hand, there were 151 DEMMRGs in GSE33479 (Figure 1B). To further narrow the key DEMMRGs in LUAD, we performed the crosstalk analysis of the differential genes between TCGA and GSE33479, thereby filtering 54 overlapped pivotal DEMMRGs (Figure 1C). To elucidate the underlying functional pathways, we conducted a KEGG enrichment analysis based on the 54 pivotal DEMMRGs. We found that the 54 genes were significantly enriched in the amino acid metabolism, vitamin metabolism, mtDNA maintenance, and Folate and 1-C metabolism pathways (Figure 1D and Supplementary File 1). These pathways were primarily involved in the activities of mitochondria, suggesting their essential roles in mitochondrial metabolism.

### Identification of PMMRGs in LUAD

**Figure 2:**
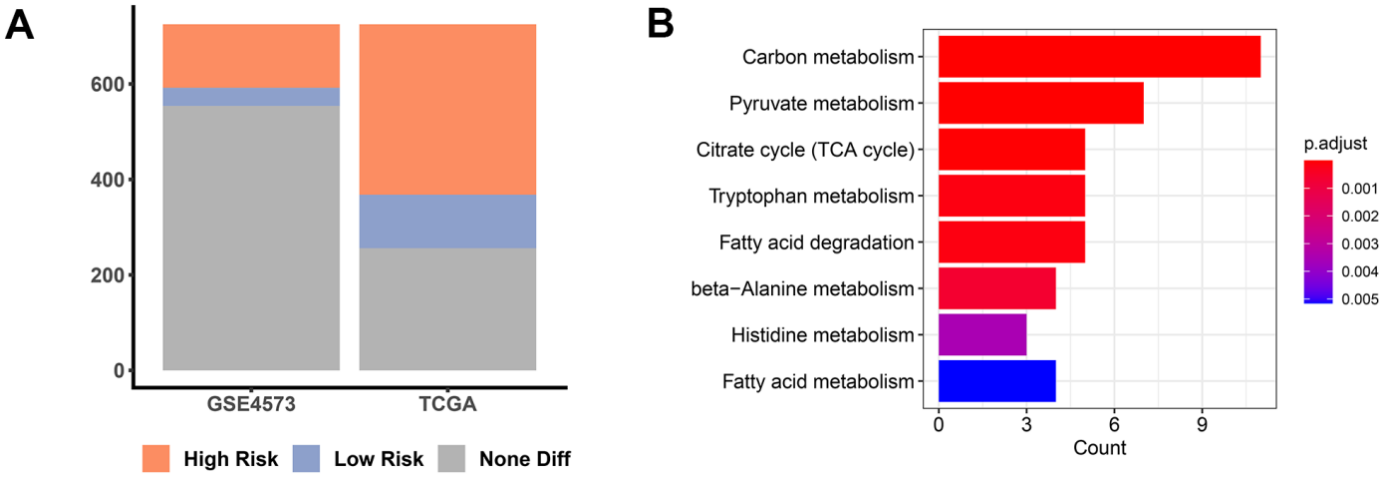
**PMMRGs identification in LUAD.** (**A**) The bar chart of prognosis-associated MMRGs in the TGCA and GSE4573 databases. (**B**) KEGG pathway enrichment analysis of 109 common MMRGs.

In an attempt to reveal the role of MMRGs during the prognosis of LUAD, we extracted the vital survival-associated MMRGs from the TCGA and GSE4573 datasets based on the 1136 genes in the MITOCARTA3.0 database. Among them, a total of 469 MMRGs were demonstrated to be related to LUAD prognosis in the TCGA database, while 171 MMRGs were found to link with LUAD survival in GSE4573 (Figure 2A and Supplementary File 2). Cross-correlation analysis showed that 109 shared MMRGs participated in the prognosis of patients with LUAD, defined as PMMRGs. KEGG enrichment analysis displayed that these PMMRGs could influence 122 pathways, wherein carbon metabolism and pyruvate metabolism were dramatically affected (Figure 2B).

### Determination of key genes in LUAD

**Figure 3:**
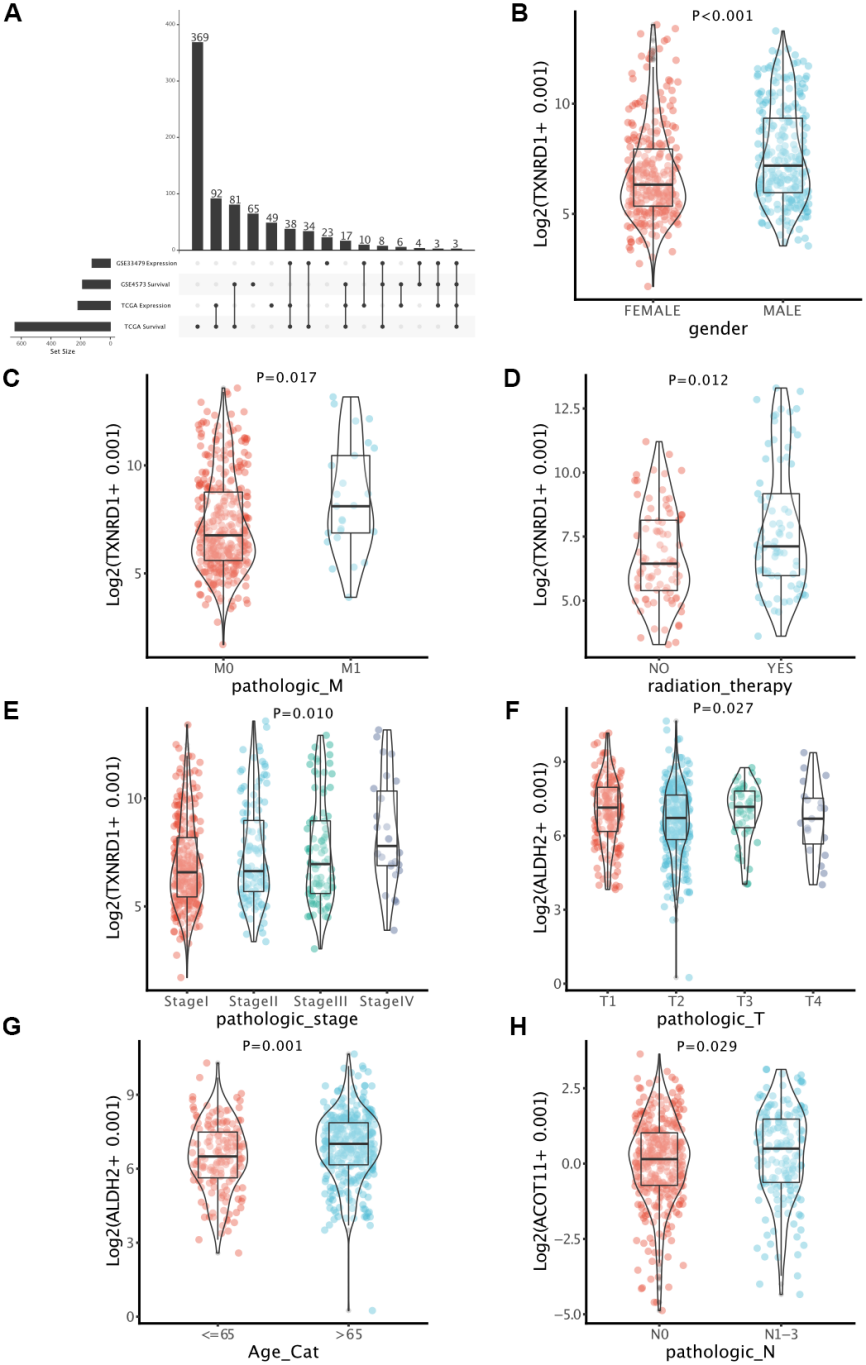
**Identification of key genes in LUAD.** (**A**) Determination of survival-associated DEMMRGs in LUAD based on four gene lists. (**B**–**H**) Boxplots of the correlation between the three genes and clinicopathological characteristics.

According to the previous four gene lists (DEMMRGs in TCGA and GSE33479, and PMMRGs in TCGA and GSE4573), we obtained three specific genes (ACOT11, ALDH2, and TXNRD1) in LUAD (Figure 3A). These key genes were involved in the progression of LUAD by affecting LUAD survival. Thus, we defined these genes as survival-related DEMMRGs. To further uncover the relationships between three survival-related DEMMRGs and clinicopathological parameter data, we delineated the landscape of three genes in age, gender, TNM stage, radiotherapy and chemotherapy situation, TNM stage, and tumor recurrence, respectively. The results manifested that TXNRD1 was associated with gender, radiotherapy receiving, and M stage (Figure 3B–3E), while ALDH2 was strongly related to T stage and age (Figure 3F, 3G). Additionally, ACOT11 was largely involved in the N stage of LUAD (Figure 3H).

### Cluster analysis identified by MMRGs

**Figure 4:**
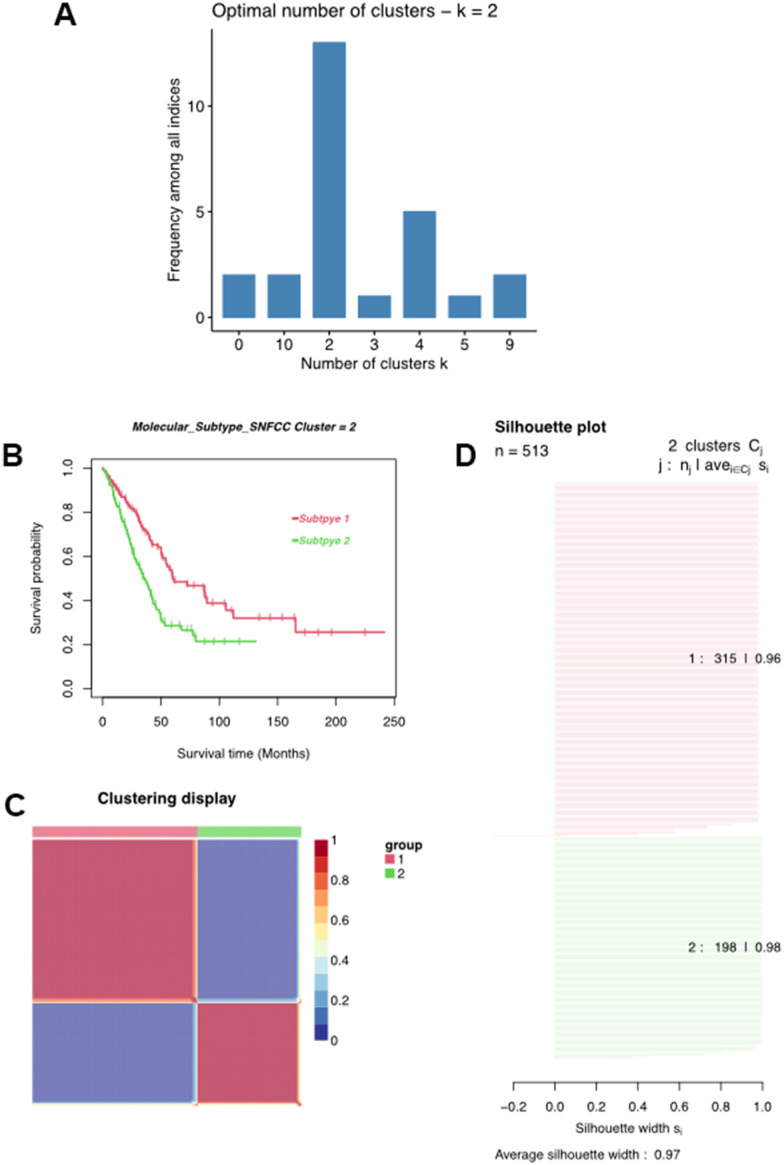
**Classification of LUAD patients.** (**A**) Multiple algorithms to determine the optimal number of clusters. (**B**) Clustering heatmap of two subtypes. (**C**) Average silhouette width exhibited the coherence of clusters. (**D**) Kaplan-Meier survival analysis of two subtypes.

Multiple algorithms were used to determine the optimal number of cluster analyses. Results indicated that more than 10 algorithms splitting the LUAD samples into two clusters were the best answer regarding this issue (Figure 4A). With this in mind, we divided these LUAD samples into Cluster1 (C1) and Cluster2 (C2) using the non-negative matrix decomposition (NMF), an effective method for clustering linearly separable data, based on the characteristics of the MMRG in LUAD [20]. Moreover, such a clustering approach could distinguish different OS of LUAD patients, as well as clear boundaries between distinct color regions, integrated with the value of average silhouette width (ASW), which is calculated as a measure of clustering consistency to estimate the degree of similarity of samples within subtypes (Figure 4C). Survival analysis indicated that the C1 cluster was negatively associated with a longer survival time in LUAD (Figure 4D).

### Clinicopathologic characteristics of the LUAD clusters

**Figure 5:**
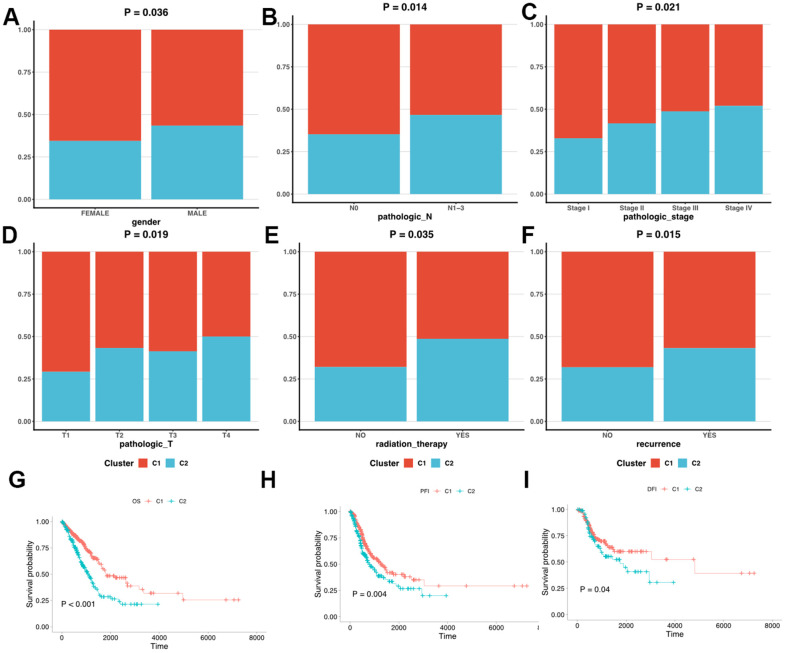
**Clinicopathologic characteristics and survival status in C1 and C2.** (**A**–**F**) The different clinicopathologic characteristics between C1 and C2. (**G**–**I**) Survival analysis of C1 and C2.

We first clarified the correlation between LUAD subtypes and clinicopathologic features and found that LUAD clusters identified by MMRGs were associated with gender, T stage, N stage, tumor recurrence, and receiving radiotherapy. (Figure 4B). Moreover, C1 displayed a weak relationship with these clinical characteristics compared to C2 (Figure 5A–5F). To further delineate the survival outcomes of LUAD patients, we carried out survival analysis in the two subtypes. Results showed that, in two clusters, there existed a difference in OS, DSS, and PFI of LUAD, suggesting the impact of LUAD clustering on LUAD survival (Figure 5G–5I).

### Functional analysis of DEMMRGs in two clusters

**Figure 6:**
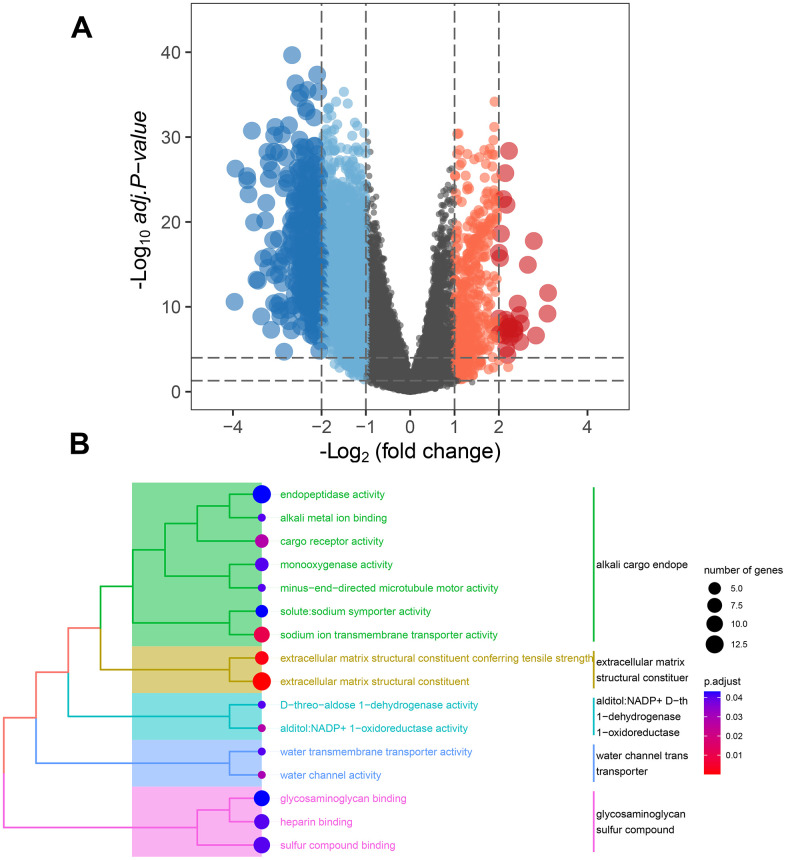
**Underlying function analysis of DEGs in two clusters identified by MMRGs.** (**A**) Volcano plot of DEGs in two clusters. (**B**) Function enrichment analysis of DEMMRGs.

In order to explore the main biological functions affected by MMRGs in the C1 and C2 clusters, we first performed the DEGs analysis by RNA-seq data from the TCGA-LUAD database between the two clusters. A total of 313 DEMMRGs were identified to be differentially expressed in the two clusters (Figure 6A). To ascertain the specific functions influenced by MMRGs, we then carried out molecular functions analysis among these DEGs. Results indicated that DEMMRGs were mainly enriched in 16 mitochondria-associated pathways (mainly were alkali cargo endopeptidase activity, extracellular matrix structural constituent, alditol: NADP+D-th1-dehydrogenase/oxidoreductase, water channel trans transporter, and glycosaminoglycan sulfur compound) (Figure 6B).

### Immune infiltration in two clusters

**Figure 7:**
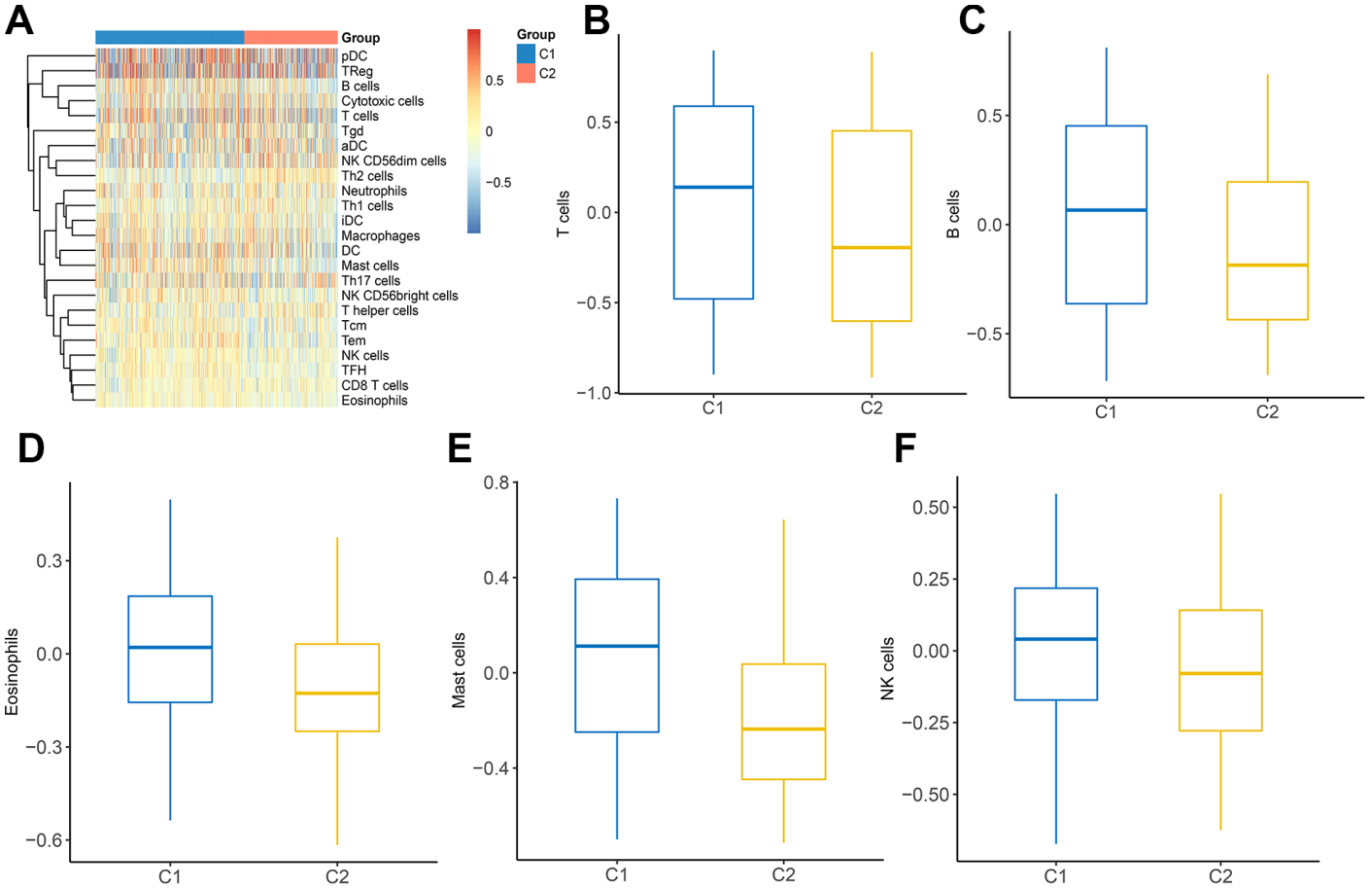
**The landscape of immune infiltration between two clusters.** (**A**) Heatmap of immune cell infiltration in two clusters. (**B**–**F**) The distribution of various immune cells in C1 and C2 was shown as a box plot.

Considering the indispensable role of immune infiltration in tumorigenesis, we analyzed the relationship between immune cells and two clusters (Figure 7A). It was observed that a variety of immune cells including T cells, B cells, eosinophils, mast cells, and NK cells, manifested a different distribution in C1 and C2 (Figure 7B–7F). These findings revealed that distinct DEGs signatures in LUAD subtypes impact immune infiltration levels.

### Tumor-specific pathways of two clusters

**Figure 8:**
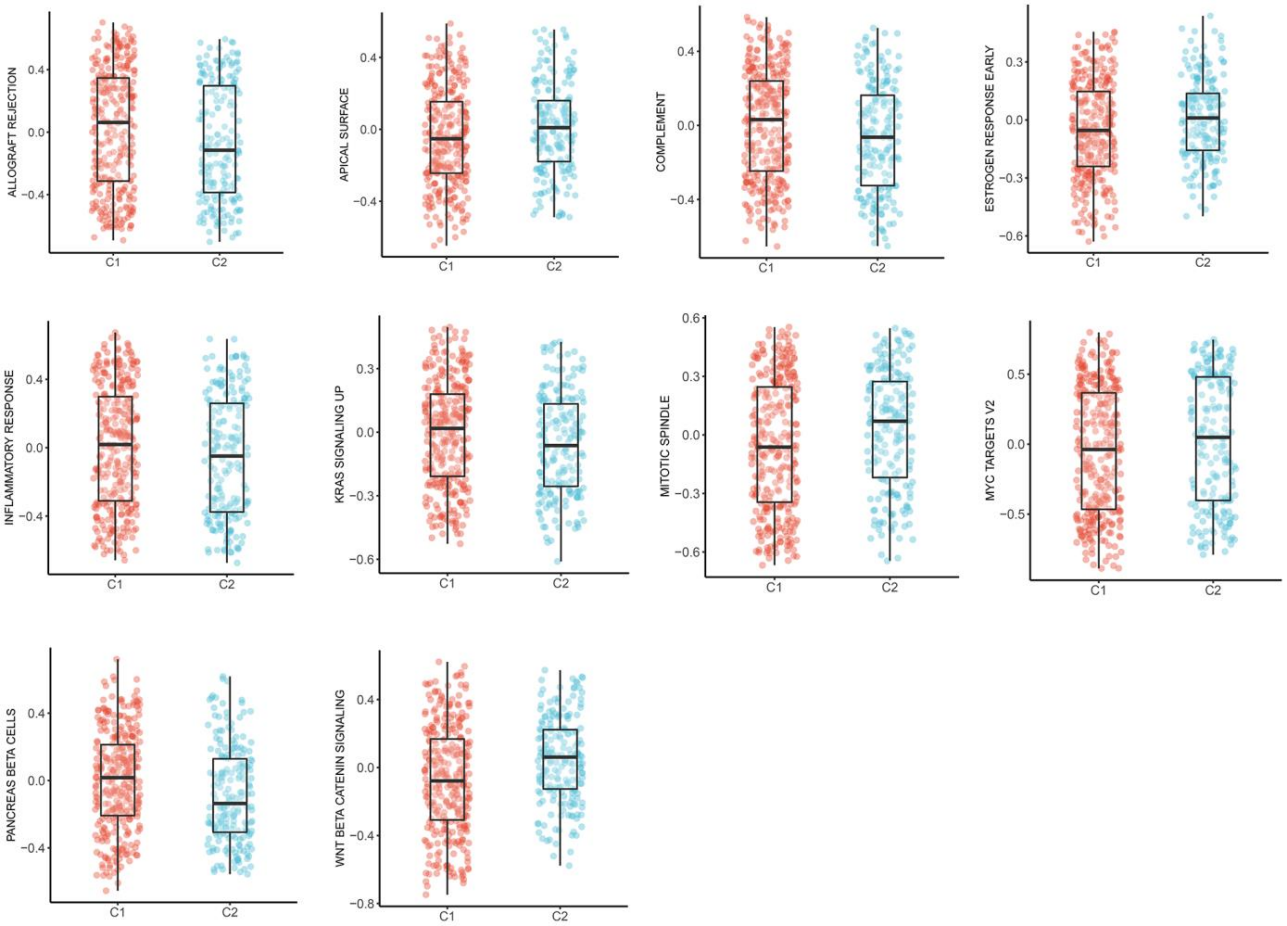
The box plots of 10 tumor-specific pathways in C1 and C2.

Expanding literature on oncology has corroborated the existence of various pathways during the development and progression of LUAD [21, 22]. Therefore, we further elucidated the main tumor-specific pathways affected by MMRGs in the well-known hallmark gene sets from GSEA analysis. Results suggested that 10 pathways, including allograft rejection, apical surface, complement, estrogen response early, inflammatory response, KAS signaling up, mitotic spindle, MYC targets V2, pancreas β cells, and Wnt/β-catenin pathways were significantly expressed in two clusters (Figure 8).

### Latent regulatory mechanisms of cluster

**Figure 9:**
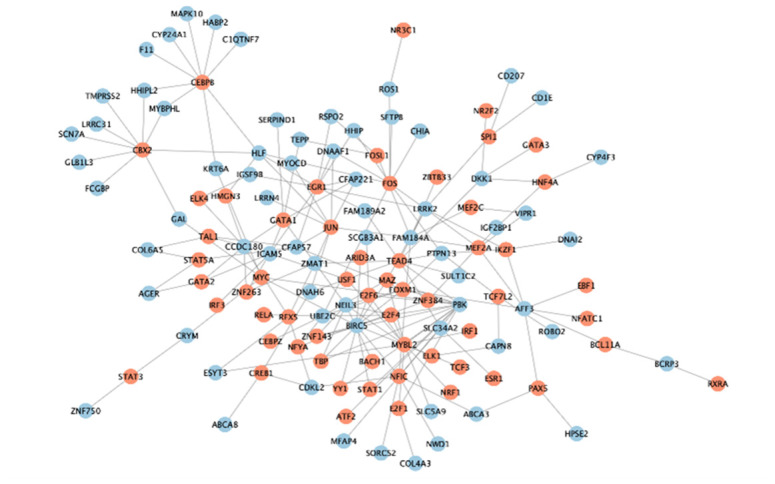
The transcriptional regulatory network of EGR1.

The previous 313 DEGs in two clusters were considered to be related to LUAD genotyping (Figure 6A). We attempted to explore the potential regulatory mechanism of these genes in LUAD progression from the perspective of gene mutation, DNA methylation, and transcription factors. Through analysis of gene mutation, we found that 198 MMRGs contained gene mutations. Among them, 25 MMRGs with gene mutations could affect the gene expression levels. 252 MMRGs were screened out to have methylation sites. Furthermore, 138 MMRGs were found to be negatively regulated by methylation sites for their innate gene expression. As for the analysis of transcription factors, 77 MMRGs were demonstrated to be manipulated by transcription factors, among which EGR1 was capable of modulating 10 MMRGs (Figure 9).

## DISCUSSION

Regardless of the tremendous progress in precision oncology through nuclear genomic analysis of LUAD, existing strategies targeting mitochondrial genetic abnormalities still exhibit limitations. A more complete understanding of LUAD based on MMRGs could fill the gap between genomic abnormalities and oncogenic MMRGs mechanisms. Mitochondria are cellular energy factories that are responsible for more than 90% of the required energy [23]. Moreover, the process of energy generation is coordinated by the interaction between nuclear and mitochondrial genomes [24]. The dysregulation and/or mutation of the mitochondrial genome exert significant effects on altering cellular metabolic status, which is permissive for tumor growth and proliferation [25]. And such characteristic metabolic transition and elevated glucose uptake in tumor cells have been previously depicted in breast cancer and pancreatic cancer [26, 27]. Also, research gives insights into the abnormality of mitochondrial energy metabolism as a vital hallmark of LUAD [28]. Given the essential role of mitochondria in metabolism, somatic mutations in the mitochondrial genome may emerge as pivotal divers of deregulated tumor cell metabolism. However, the mutational profile of the mitochondrial genome has not been extensively studied. Here, we identified key MMRGs in LUAD progression and prognosis and as well as their potential functional mechanisms.

Firstly, 1136 MMRGs were obtained from MITOCARTA3.0. Then, differential expression analysis was exploited to select DEMMRGs from the TCGA and GSE33479 datasets, and 54 DEMMRGs were ultimately identified. Subsequently, we found that these 54 genes were mainly engaged in amino acid metabolism, vitamin metabolism, mtDNA maintenance, and folate and 1-C metabolism pathways (Figure 1B). These metabolism-associated pathways showed great promise for clinical use. For example, glutaminolysis is therapeutically exploited in subsets of KRAS-mutant LUAD through glutaminase inhibition. KRAS is one of the most commonly mutated oncogenic drivers in LUAD that has yet to be fully conquered in cancer treatment given the challenge of inhibiting KRAS directly [29]. However, mutations that frequently co-occur with those in KRAS could be defined as therapeutic vulnerabilities in LUAD. In KRAS-mutant LUAD, tumors with LKB1 loss are highly enriched for concurrent KEAP1 mutations, which activate the KEAP1/NRF2 pathway [30]. LKB1-deficient tumors were dramatically concentrated with concurrent KEAP1 mutations, which in turn activated the KEAP1/NRF2 signaling pathway. Initiation of the KEAP1/NRF2 axis in tumors with LKB1 loss increased cell survival rates and maintained the energetic and redox homeostasis in a glutamine-dependent manner [31].

Subsequently, based on the TGCA and GSE4573 databases, 109 common PMMRGs were collected and they were involved in 122 pathways, such as carbon metabolism and pyruvate metabolism, which were crucial in mitochondria. Combining the differential expression profiles and survival analysis of MMRGs in LUAD, three MMRGs (ACOT11, ALDH2, and TXNRD1) were identified as prognostic DEMMRGs. ACOT11 encoded enzymes that participated in the metabolism of fatty acids [32, 33]. We found that ACOT11 was correlated to the N stage. An isolated study revealed that ACOT11 was upregulated in LUAD patients and generally associated with an unfavorable prognosis, which was in accord with our study [34]. ACOT11 could regulate cell proliferation, migration, and invasion of LUAD through multiple signaling pathways, suggesting its promising potential in molecular treatment [34]. ALDH2 is capable of detoxifying acetaldehydes into non-toxic acetic acids [35]. Our study showed that ALDH2 was associated with age and T stage. It was reported that ALDH2 repression contributed to a dismal prognosis of patients with LUAD [36]. As for TXNRD1, there were few studies focused on its role in LUAD. However, in our current study, TXNRD1 was discovered to be related to gender, receiving radiotherapy, and the M stage. Taken together, our research extended the current knowledge by emphasizing the scientific merits of these three PMMRGs during LUAD progression.

Further, to get a more comprehensive landscape of MMRGs in LUAD, we divided them into two clusters (C1 and C2) and explored the difference of clinicopathological features in C1 and C2. The results manifested that C2 had more apparent characteristics compared with C1. Furthermore, the MMRGs-signature influences the OS, DSS, and PFI of LUAD clusters. 313 differentially expressed MMRGs are identified in C1 and C2 and they were mainly enriched in 16 methodical-associated function pathways, in particular, extracellular matrix (Figure 6B). Indeed, it has been established that the tumor extracellular matrix was responsible for drug resistance and immune suppression [37]. Then, we investigated the context of immune cell infiltration level in LUAD given the increasingly important role of immune infiltration in tumorigenesis [38]. There existed a clear difference in the distribution of various immune cells in C1 and C2. In addition, the pathways influenced by MMRGs were delineated. 10 characteristic pathways were ultimately figured out, such as Wnt/β-catenin signaling, which was a critical driver in epithelial-mesenchymal transition and tumor metastasis [39].

Finally, the detailed mechanisms underlying the MMRGs signatures in two clusters were investigated from the perspective of gene mutation, DNA methylation, and transcription factors. In general, DNA methylation is more frequent in solid tumors compared with genomic mutations [40]. Both hypermethylation of tumor suppressor genes and hypomethylation of carcinogenic genes are essential factors in tumor development and progression. However, few studies have been conducted to decode the function of MMRGs in DNA methylation, which may be an important aspect to focus on in future research. More importantly, in LUAD progression, we identified a key transcription factor of MMRGs clustering, EGR1, which modulated 10 genes. As a transcriptional activator, EGR1 has been proposed to induce overexpression of LINC01116 [41]. However, the exact function of EGR1 in LUAD still needs more assays to illustrate.

## CONCLUSIONS

In summary, we provided a complementary and more comprehensive understanding of MMRGs during the development and progression of LUAD. Also, we investigated the latent mechanisms underlying the MMRGs in LUAD. These findings may offer an opportunity to expedite translation of basic research to more precise treatment in the clinic.

## Supplementary Material

Supplementary File 1

Supplementary File 2
